# The impact of PARPs and ADP-ribosylation on inflammation and host–pathogen interactions

**DOI:** 10.1101/gad.334425.119

**Published:** 2020-03-01

**Authors:** Anthony R. Fehr, Sasha A. Singh, Catherine M. Kerr, Shin Mukai, Hideyuki Higashi, Masanori Aikawa

**Affiliations:** 1 Department of Molecular Biosciences, University of Kansas, Lawrence, Kansas 66045, USA;; 2 Center for Interdisciplinary Cardiovascular Sciences, Cardiovascular Division, Brigham and Women's Hospital, Harvard Medical School, Boston, Massachusetts 02115, USA;; 3 Center for Excellence in Vascular Biology, Brigham and Women's Hospital, Harvard Medical School, Boston, Massachusetts 02115, USA;; 4 Department of Human Pathology, I.M. Sechenov First Moscow State Medical University of the Ministry of Health, Moscow 119146, Russian Federation

**Keywords:** ADP-ribosylation, PARP, atherosclerosis, host–pathogen interactions, immunity, inflammation, macrophage, vascular disease

## Abstract

Poly-adenosine diphosphate-ribose polymerases (PARPs) promote ADP-ribosylation, a highly conserved, fundamental posttranslational modification (PTM). PARP catalytic domains transfer the ADP-ribose moiety from NAD^+^ to amino acid residues of target proteins, leading to mono- or poly-ADP-ribosylation (MARylation or PARylation). This PTM regulates various key biological and pathological processes. In this review, we focus on the roles of the PARP family members in inflammation and host–pathogen interactions. Here we give an overview the current understanding of the mechanisms by which PARPs promote or suppress proinflammatory activation of macrophages, and various roles PARPs play in virus infections. We also demonstrate how innovative technologies, such as proteomics and systems biology, help to advance this research field and describe unanswered questions.

## Abstract (precis)

In this review, Fehr et al. summarize the current understanding of the mechanisms by which PARPs promote or suppress proinflammatory activation of macrophages, and also discuss various other roles PARPs play in virus infections.

Polyadenosine diphosphate-ribose polymerases (PARPs) promote ADP-ribosylation, one of the fundamental posttranslational modifications (PTMs) (Gupte et al. 2017). This ubiquitous PTM regulates various key biological and pathological processes, including DNA repair, cell differentiation, gene transcription, signal transduction pathways, energy metabolism, and epigenetics. PARP catalytic domains transfer the ADP-ribose moiety from NAD^+^ to amino acid residues of target proteins, leading to mono- or poly-ADP-ribosylation (MARylation or PARylation). PARP members thus function as “writers” of ADP-ribose. Among the 17 human PARPs, PARP1, PARP2, PARP5A, and PARP5B promote PARylation, while most other members (e.g., PARP3, PARP4, PARP6, PARP14, and PARP15) catalyze MARylation (Hottiger 2015; Ryu et al. 2015; Gupte et al. 2017). A new nomenclature has thus been proposed to call them the diphtheria toxin-like ADP-ribosyltransferases (ARTDs); e.g., ARTD1 for PARP1 (Hottiger et al. 2010). Differences between each PARP lead to diverse functions for PARPs in biological processes such as the innate immune response (Fig. 1).

**Figure 1.**
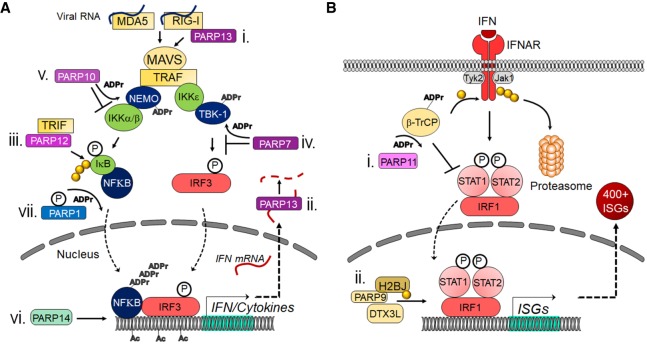
PARPs regulate the innate immune response at many different points. (*A*) Mechanisms used by MARylating and nonenzymatic PARPs to modulate IFN and proinflammatory cytokine induction. (i) PARP13 can bind to RIG-I, which promotes its oligomerization and the initiation of the cascade. (ii) PARP13 can also bind to IFN mRNA and target it for degradation. (iii) PARP12 was shown to bind TRIF and enhance NFκB-dependent gene expression. (iv) PARP7 can ADP-ribosylate TBK-1, which inhibits it from phosphorylating IRF3. (v) PARP10 can interact with and ADP-ribosylate NEMO, which prevents the activation of IKKs. (vi) PARP14 promotes H3K27 acetylation and recruitment of Pol II to IFN promoters. (vii) Upon phosphorylation, PARP1 can poly-ADP-ribosylate NFκB and promote its activity. (*B*) Mechanisms used by MARylating and nonenzymatic PARPs to modulate IFN-I signaling. (i) PARP11 binds to and ADP-ribosylates the E3 ubiquitin ligase β-TrCP. This allows β-TrCP to interact with and ubiquitinate IFNAR, which targets it for proteasome-dependent degradation. (ii) PARP9 and DTX3L interact with and ubiquitinate histone protein H2BJ, which leads to chromatin remodeling that enhances expression of a subset of ISGs. (P) Phosphate group; (ADPr) ADP-ribose; (Ac) acetyl modification; (yellow ciricle) ubiquitin.

PARP family members contain a few structural domains, in addition to the catalytic domain. One of such domains is the macrodomain that is contained in PARP9, PARP14, and PARP15, for which they are called “macro” PARPs. Macrodomains bind to, and in some cases hydrolyze, ADP-ribose in the free or protein-bound form (“readers” of ADP-ribosylation) and influence many biological processes (Rack et al. 2016). Evidence has linked the MacroPARPs PARP9 and PARP14 in multiple types of cancers, particularly lymphomas (Aguiar et al. 2000; Cho et al. 2009). PARP14 may also play an important role in cell morphology (Vyas et al. 2013). We found the interplay of PARP9 and PARP14 in the regulation of macrophage activation (Iwata et al. 2016), as described in this review.

Different cellular distributions of PARPs may also indicate their distinctive targets and functions (Vyas et al. 2013). While PARP1 is only found in the nucleus, PARP6, PARP8, PARP12, PARP13, PARP15, and PARP16 are mostly located in the cytoplasm. PARP2, PARP3, PARP4, PARP5A, PARP5B, PARP7, PARP9, PARP10, PARP11, and PARP14 are seen in both the nucleus and cytoplasm.

ADP-ribolylation is reversed by “erasers” such as poly-ADP-ribose glycohydrolase (PARG), ADP-ribosylhydrolase 3 (ARH3), and macrodomains such as Mdo2 (Miwa and Sugimura 1971; Oka et al. 2006; Jankevicius et al. 2013; Rosenthal et al. 2013). PARG is a potent enzyme that degrades poly-ADP-ribose, with several isoforms that are derived from the single PARG gene through alternative splicing (i.e., 110-, 102-, 99-, and 60-kDa proteins). The 110-kDa isoform, mostly seen in the nucleus, appears to play a dominant role in the PAR degradation. PARG cannot erase ADP-ribose when bound to proteins and leaves MARylated amino acid residues. PARG is a useful tool that enables researchers the ability to enrich for MARylated peptides for mass spectrometry analysis of ADP-ribosylation (Higashi et al. 2019).

## PARPS in immune cells: a focus on inflammation

Immune cells serve an important role in the immune system and differentiate into various subsets that perform a spectrum of unique functions. The balance of the number of different immune cell types and their activation levels is crucial for health and disease. Overwhelming evidence has associated chronic inflammation with various pathological conditions and their potential causes, including atherosclerosis, cardiovascular events, cancer, autoimmune diseases, metabolic disorders, neurological diseases, and aging (Johnston et al. 1987; Gisterå and Hansson 2017; Tabas and Lichtman 2017; de Vries and Quax 2018; Gomez et al. 2018; Aday and Ridker 2019; Bercovici et al. 2019; Di Benedetto et al. 2019; Guner and Kim 2019; Horwitz et al. 2019; O'Rourke et al. 2019; Othman et al. 2019; Trott and Fadel 2019). Many investigations have focused on the major role of macrophages in such contexts and mechanisms for their proinflammatory activation (Murray and Wynn 2011; Wynn and Vannella 2016; Gisterå and Hansson 2017; Tabas and Lichtman 2017; Decano and Aikawa 2018; Funes et al. 2018; Swirski and Nahrendorf 2018; O'Rourke et al. 2019). Various signal-transduction pathways participate in macrophage activation, which are often regulated by PTMs such as phosphorylation and acetylation (Tietzel and Mosser 2002; Park et al. 2011; Zhou et al. 2014; Nakano et al. 2016; Vergadi et al. 2017; Dean et al. 2019). This section focuses on the impact of PARPs and ADP-ribosylation in macrophage activation and also summarizes their roles in the biology of other immune cells.

### PARP1 induces macrophage activation and inflammation

Evidence suggests that ADP-ribosylation participates in inflammation (Bai and Virág 2012; Rosado et al. 2013; Kunze and Hottiger 2019). PARP1 has been implicated in the mechanisms for responses (e.g., proinflammatory cytokine expression) of macrophages or macrophage-like cell lines to pathogen-associated molecular patterns (PAMPs), including lipopolysaccharide (LPS) (Hassa et al. 2005; Liu et al. 2012a; Yang et al. 2014; Minotti et al. 2015; Bohio et al. 2019). Some responses involve the interplay between PARP1 and nuclear factor κB (NF-κB), a key transcription factor in immunity and various other biological processes (Hassa and Hottiger 1999; Hassa et al. 2005; Liu et al. 2012a; Minotti et al. 2015; Bohio et al. 2019; Kunze and Hottiger 2019). Using PARP1-deficient mice Oliver et al. (1999) provided evidence that PARP1 promotes NF-κB activation in macrophages in vivo. This study demonstrated that *Parp1* deletion causes resistance to LPS-induced endotoxic shock by NF-κB-dependent iNOS induction and NO production. Recent evidence suggests that phosphorylation of PARP1 results in PARylation of the NF-κB subunit p65/RelA, which induces the transcription of NF-κB-regulated genes (Fig. 1A; Bohio et al. 2019). PARP1 also induces the release of the high-mobility group box 1 (HMGB1), a proinflammatory factor, from the nucleus to the cytoplasm in macrophages, which requires its PARylation and subsequent acetylation (Ditsworth et al. 2007; Yang et al. 2014). PARP1 also exerts proinflammatory effects on other macrophage-related cell types such as Kupffer cells in the fatty liver and microglia in the injured brain (Ullrich et al. 2001; Mukhopadhyay et al. 2017). In some cases, PARP-1-induced activation of proinflammatory mediators, such as NF-κB, do not depend on its enzymatic activity, suggesting mechanisms used by PARP1 to impact inflammation depend on the context or its targets (Hassa et al. 2005; Minotti et al. 2015).

### The role of nicotinamide adenine dinucleotide (NAD^±^) in PARP1-mediated macrophage activation

PARPs catalyze the transfer of ADP-ribose from NAD^±^ to target proteins. Hence, NAD^±^ is consumed by PARPs, and the activity of PARPs depends on the availability of NAD^±^ (Gupte et al. 2017). A recent report indicates that cell-autonomous production of NAD^±^ via the kynurenine pathway (KP) is required to induce normal inflammatory macrophage activation and that the de novo NAD^±^ synthesis can be impaired in aged macrophages (Minhas et al. 2019). Another study proposed a mechanism linking the NAD^±^ salvage pathway to LPS-induced PARP1 consumption of NAD^±^ (Cameron et al. 2019). In LPS-stimulated macrophages, an increase in reactive oxygen species induces DNA damage, which in turn activates PARP1, leading to a reduction of available NAD^±^. Nicotinamide phosphoribosyltransferase (NAMPT) is therefore increased to maintain NAD^±^ levels, which is crucial for normal inflammatory macrophage activation.

### PARP1 participates in the biology of other immune cells

PARP1 modulates the differentiation of T cells into effector T cells such as T helper 1 (Th1), T helper 2 (Th2), and regulatory T cells (Tregs) (Saenz et al. 2008; Nasta et al. 2010). PARP1 deficiency in murine T cells leads to the increased expression of the Th1 cytokine interferon-γ (IFN-γ) and the decreased production of the Th2 cytokine interleukin 4 (IL-4) (Saenz et al. 2008). IL-4 suppresses IFNγ secretion and Th1 differentiation, and PARP1 promotes IL-4 expression via chromatin modifications at the IL-4 locus (Saenz et al. 2008). Although PARP1 is not involved in the differentiation of naïve T cells into T helper 17 (Th17) cells, it does impact Tregs, as these cells are augmented in multiple organs in PARP1-deficient mice (Nasta et al. 2010). Using PARP1-deficient mice, Nasta et al. (2010) demonstrated that PARP1 supresses the expression of Foxp3 and thus generation of Tregs via modulation of the chromatin structure and/or regulation of the transcription factors. Additional studies used PARP1-deficiet mice further demonstrated mechanisms for PARP1-regulated suppression of Tregs via transforming growth factor β (TGF β) receptors (Zhang et al. 2013). Recent reports demonstrate the interactive role of PARP1 and PARP2 in maintaining the number and function of T cells and promoting the development and function of B cells (Navarro et al. 2017; Galindo-Campos et al. 2019). Defective thymocyte maturation is observed in PARP1/PARP2-deficient mice, and accordingly T-cell numbers in peripheral blood are reduced (Galindo-Campos et al. 2019). In PARP1/PARP2-deficient mice, the development of bone marrow B cells is impaired, leading to the reduction of transitional and follicular B cells (Navarro et al. 2017). PARP1 also plays a role in the maturation and function of dendritic cells by regulating the production of IL-10 and IL-12 (Aldinucci et al. 2007).

### PARP1 promotes experimental cardiovascular disorders

A series of in vivo studies from the Boulares and Matter groups (Oumouna-Benachour et al. 2007; von Lukowicz et al. 2008; Hans et al. 2009, 2011) used PARP1-deficient mice to demonstrate that PARP1 promotes the development of various cardiovascular disorders. Two studies reported that PARP1 deficiency in apolipoprotein E-deficient (*ApoE*^−/−^) mice reduces the size, macrophage and T-cell content, death of macrophage foam cells, necrotic core, NF-kB activation, and adhesion molecule expression in experimental atherosclerotic lesions, typical features of human plaques prone to acute thrombotic events (Oumouna-Benachour et al. 2007; von Lukowicz et al. 2008). *Parp1* deletion attenuates dyslipidemia-induced vascular dysfunction in *ApoE*^−/−^ mice seemingly via maintenance of eNOS activity (Hans et al. 2011). PARP1 deficiency furthermore improves delated cardiomyophathy and concomitantly increases tissue inhibitor of metalloproteinase 2 (TIMP2) in *ApoE*^−/−^ mice (Hans et al. 2009).

### PARP9 and PARP14 regulate macrophage activation

While many reports suggested multiple proinflammatory roles for PARP1, contributions of other PARP family members in macrophage activation remain incompletely understood. We demonstrated that PARP9 and PARP14 coregulate proinflammatory activation of human macrophages (Iwata et al. 2016). In this study we took a systems-biology approach involving unbiased proteomics, bioinformatics, and network analysis to identify potential molecular switches of the balance of proinflammatory versus non/anti-inflammatory macrophage phenotypes as potential therapeutic targets. We performed proteomics of human and mouse macrophage-like cell lines treated with IFNγ or IL-4, which represent so-called proinflammatory M1 versus non/anti-inflammatory M2 cells. We processed our proteomics data of >5000 proteins with a conventional filtering method as well as our original clustering method (Ricchiuto et al. 2015) to identify molecules that increased with IFNγ and decreased with IL-4 (Iwata et al. 2016). Interestingly, the only protein that emerged from this stringent criteria was PARP14. We also recognized that PARP9 showed similar responses.

**Figure 2.**
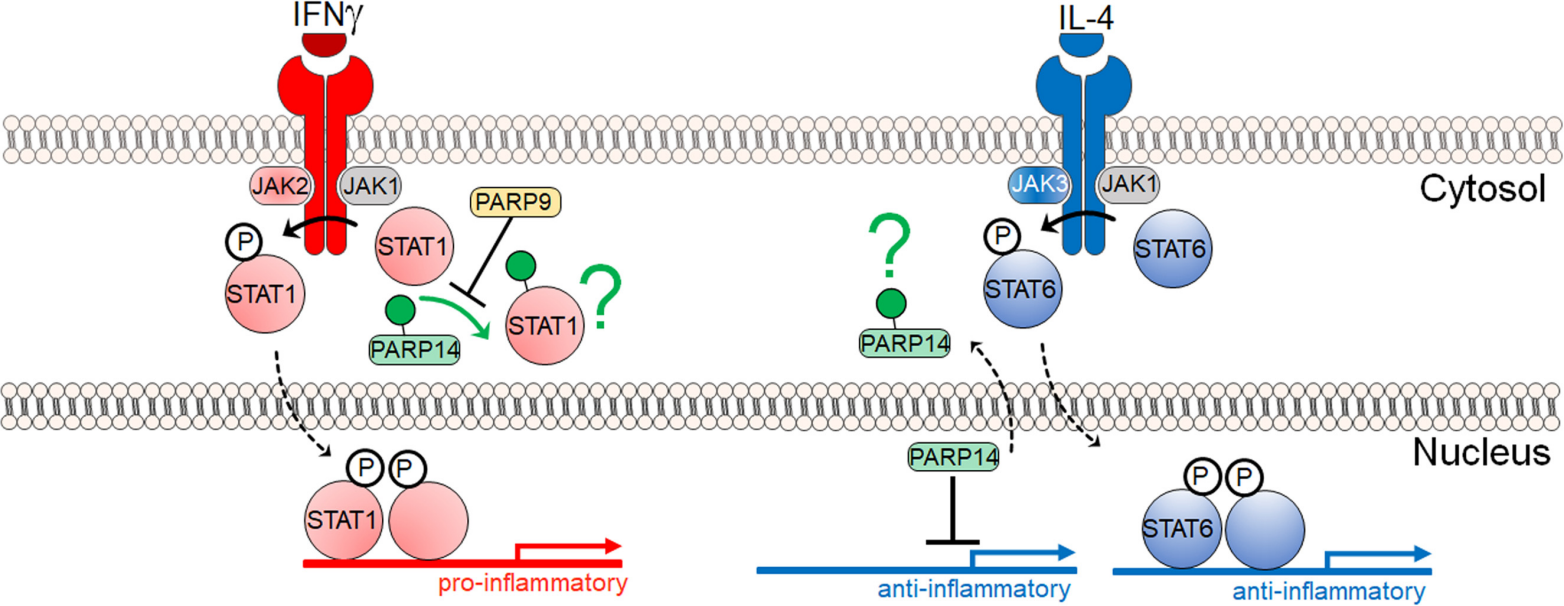
A partial model of PARP14 and PARP9 function in macrophage activation. In vivo and in vitro studies pertaining to IFNg signaling in primarily macrophages suggest that PARP14 mitigates proinflammatory phosphorylated STAT1 via ADP-ribosylation, and that PARP9 may act to inhibit PARP14's enzymatic activity (Iwata et al. 2016). In vitro studies pertaining to IL-4 signaling in the context of B-cell biology suggest that in nonstimulating conditions PARP14 is a suppressor of STAT6 target genes. In response to IL-4, PARP14 is thought to become enzymatically active and dissociate from the promoter(s), thereby allowing phosphorylated STAT6 to bind and activate target genes (Mehrotra et al. 2011). A green question mark indicates that the fate of ADP-ribosylated substrates is not known. The IFNγ and IL-4 mechanisms appear distinct, but they may be partial and complementary pictures of a complex biology.

The same study demonstrated in vitro that PARP14 suppresses proinflammatory IFNγ–STAT1 signaling and activates the anti-inflammatory IL-4-STAT6 pathway in primary human macrophages (Fig. 2; Iwata et al. 2016). Silencing of PARP14 by siRNA accelerated the induction of proinflammatory cytokines and chemokines (e.g., TNFα, IL-1β, and CCL2/MCP-1) in IFNγ-treated macrophages and suppressed anti-inflammatory molecules (e.g., MRC1and Arg1) in IL-4-treated cells. PARP9 silencing generally exerted opposing effects. PARP9 also appeared to interfere with PARP14's suppressive action on the IFNγ–STAT1 axis, thus promoting proinflammatory macrophage activation (Fig. 2). Cell-free enzyme reactions with mass spectrometry as a read-out further indicated that ADP-ribosylation of STAT1 by PARP14 may reduce phosphorylation of this proinflammatory mediator. However, the mechanisms used by PARP14 to interact with STAT1 and influences its ADP-ribosylation and phosphorylation requires further investigations.

**Figure 3.**
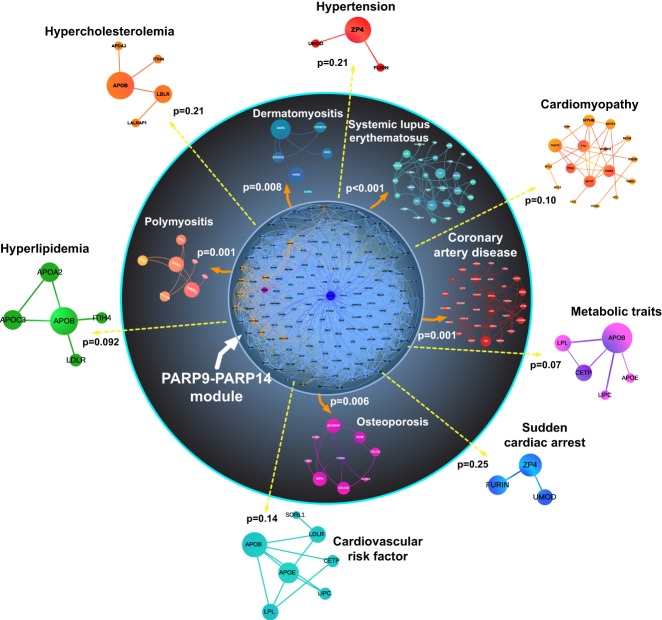
Computational prediction of an association between the PARP9–PARP14 network and human inflammatory diseases. The network of PARP14 (blue)–PARP9 (purple) consists of proteins that directly interact with these PARPs (blue and orange nodes, respectively). *P*-values indicate the significance of closeness between the PARP14–PARP9 first neighbors in the interactome (the PARP9–PARP14 module) and gene modules of human diseases such as coronary artery disease compared with random expectation. Reproduced from Iwata et al. (2016).

Due to the well-recognized knowledge that targets identified in basic science often fail in the clinical stage, we performed network analysis that closely linked the network of PARP9, PARP14, and their first neighbor interactors with the human coronary artery disease gene module (Fig. 3). As predicted, in vivo studies in *Parp14*^−/−^ mice demonstrated that PARP14 participates in the pathogenesis of arterial diseases. Consistent with in vitro studies, PARP14 deficiency indeed mitigated lesion development and inflammatory burden in models of coronary artery disease in mice (Iwata et al. 2016).

The anti-inflammatory role of PARP14 reported by us are consistent with other reports. Iqbal et al. (2014) demonstrated in macrophages that PARP14 reduces mRNA stability and thus expression levels of tissue factor, a surface glycoprotein that plays a major thrombogenic role in macrophage-rich atherosclerotic lesions. PARP14's association with anti-flammatory STAT6 was first described using a yeast two-hybrid screen (Goenka and Boothby 2006). Although subsequent studies were not done in the context of macrophage activation, they indicated that PARP14's enhancement of IL-4 STAT6's transcriptional activity may be more relevant to its promoter binding functions rather than its potential to ADP-ribosylate STAT6 itself (Fig. 2; Goenka et al. 2007; Mehrotra et al. 2011). For instance, PARP14 can act as a transcriptional repressor of STAT6 target genes, but activation by IL-4 leads to its autoribosylation and dissociation of PARP14 from a DNA-protein complex, thereby promoting STAT6 binding instead (Fig. 2; Goenka et al. 2007; Mehrotra et al. 2011). Thus, despite the accumulating evidence that PARP14 promotes an anti-inflammatory state, there does not yet exist enough information to disentangle PARP14's enzymatic functions from that of its protein-interaction functions, and how each of these functions may maintain an anti-inflammatory response, irrespective of the cytokine or stimulus.

Evidence established that macrophages are a heterogeneous group of cells, as represented by the well-known theory of M1 versus M2 polarization. Recent understanding, however, suggests that macrophage heterogeneity is more complex and multidimensional than the M1/M2 dichotomy (Murray et al. 2014; Nahrendorf and Swirski 2016; Decano and Aikawa 2018). In our study, single-cell analysis demonstrated that IFNγ-elicited macrophages remain largely heterogeneous, consisting of a few clusters rather than uniformly “polarized” toward an activated phenotype (Iwata et al. 2016). Gene similarity maps demonstrated close interactions between PARP9, PARP14, STAT1, and STAT6, supporting our in vitro data described above.

### PARP14 regulates lymphocyte biology

Several pieces of evidence demonstrate that PARP14 promotes the differentiation of naïve T cells into Th2 cells by regulating STAT6-dependent *Gata3* expression and that PARP14-deficient mice show reduced symptoms of allergic airway disease (Mehrotra et al. 2013; Riley et al. 2013). PARP14 enhances STAT3-dependent Th17 differentiation (Mehrotra et al. 2015). PARP14 is also implicated in immunoglobulin class switching in B cells by enhancing the IL-4 and STAT6 signal, which produces the IgE isotype, a major factor in allergic hypersensitivity (Mehrotra et al. 2011).

### Other PARPs in macrophage biology

As discussed, while several studies have reported how PARP1, PARP2, PARP9, and PARP14 promote or suppress macrophage activation via signaling pathways (e.g. NF-κB, IFNγ–STAT1, and IL-4–STAT6), the evidence remains scant on the roles that other PARPs play in macrophage biology. LPS increases the mRNA expression of PARP3, PARP4, PARP7, PARP8, PARP10, PARP11, PARP12, and PARP13 in murine bone marrow-derived macrophages, but there are no known functions for these PARPs in macrophage biology (Caprara et al. 2018). Other reports have linked PARP10 and PARP12 with NF-κB signaling (Verheugd et al. 2013; Welsby et al. 2014). Although these lines of evidence may suggest roles of PARPs other than PARP1, PARP2, PARP9, and PARP14 in macrophage biology, more investigations are needed to better understand how these PARPs participate in macrophage activation and inflammatory diseases.

### PARPs in cancer immunology

PARPs have been targets for drug development, particularly in the cancer field. The most active targets include PARP1, and more recently PARP14 (Berger et al. 2018; Qin et al. 2019). Studies that used the small molecule inhibitors demonstrated that PARP suppression reduces proinflammatory responses or enhances anti-inflammatory functions of macrophages (Haskó et al. 2002; Wang et al. 2013; Shrestha et al. 2016; Dharwal et al. 2019). Recent work showed that PARP inhibition in combination with PD-1/PD-L1 blockade could be effective for BRCA1-deficient tumors by activating antigen presenting cells such as dendritic cells via the cGAS-STING pathway (Jiao et al. 2017; Ding et al. 2018; Dunphy et al. 2018). Major effects reported in these studies may reflect PARP1 suppression. These inhibitors, however, are not strictly specific for PARP1 and their effects on other PARP members and targets other than PARPs thus need to be addressed (Wahlberg et al. 2012).

## Activated macrophages link ADP-ribosylation with protein homeostasis

A global view of PARP activity on immunity undoubtedly requires proteomics. However, even with the most advanced mass spectrometry technologies available, disentangling the unique and overlapping functions of PARPs remains very challenging. Only a handful of studies have begun to interrogate the complex biology of the PARP family members in the context of immunity.

Nearly 30 years ago, radioactive labeling strategies demonstrated an increase in ADP-ribosylation signal in human monocyte-derived macrophages in response to IFNγ (Berton et al. 1991). In addition, there was no increase in PARP1 mRNA, implicating additional mechanisms rather than an increase in total PARP1 levels for the increase in ADP-ribosylation. Using quantitative proteomics, we made a similar observation in IFNγ-treated THP-1 and RAW264.7 macrophage-like cell lines (Iwata et al. 2016). Moreover, PARP14 and PARP9 exhibited the increased expression following IFNγ exposure and had the opposite response to IL-4, decreasing in abundance over the stimulation period. These contrasting proinflammatory and anti-inflammatory responses by PARP9 and PARP14 were distinct from the other PARPs measured in the study. More specifically, their responses to IFNγ were characteristic of known cytokine inducible genes, STAT1, NMI (N-myc interactor), OAS2, IFIH1, and IFIT3, among several others. This study thus provided a plausible scenario where the increase in global ADP-ribosylation in macrophages could be mediated in part by PARP14 (Iwata et al. 2016).

In a separate study, using quantitative proteomics in combination with shRNA-mediated knockdown experiments, PARP14 interactors were pursued in the context of LPS signaling in RAW264.7 cells (Caprara et al. 2018). A PARP14 coimmunoprecipitation of these RAW264.7 cells after LPS treatment found that SQSTM1 (Sequestome-1, a receptor linking autophagy and ubiquitylation), PARP9, DTX3L, and NMI were immunoprecipitated with a PARP14 antibody specifically in PARP14 wild-type but not PARP14 knockdown cells, indicating that these proteins form a PARP14–protein interaction network in response to LPS.

Protein–protein interactions alone do not clarify the role of PARP enzyme activities in immunity. To date, only one study has investigated ADP-ribosylated substrates in immune cells on a global level (Higashi et al. 2019). Since ADP-ribosylome studies are technically challenging to perform; they rely on specialized proteomic workflows to enrich and sequence ADP-riboyslated peptides (Martello et al. 2016; Larsen et al. 2017). We used two independent approaches to enrich the ADP-ribosylome of IFN-γ-treated THP-1 cells. One approach used an anti-PARylation antibody enrichment that provided candidate ADP-ribosylated proteins, and the other enriched for MARylated peptides via their affinity for a macrodomain to identify ADP-ribosylated proteins (Higashi et al. 2019). The majority of proteins identified from both approaches combined, comprised ribosome/RNA-binding proteins. These findings were not surprising since this protein class is highly abundant, and thereby more conducive to identifying ADP-ribosylated peptides. Other ADP-ribosylated proteins were associated with pathways involved in neutrophil degranulation and activation, IL-12 signaling, and glycolysis. Moreover, ADP-ribosylation of a subset of the ribosome/RNA-binding proteins increased in response to IFNγ, as did ADP-ribosylated forms of PARP14 and PARP9, and interestingly SQSTM1, the ubiquitin pathway associated protein identified as a candidate PARP14 binding partner (Caprara et al. 2018). These studies point toward ADP-ribosylation linking macrophage activation with protein homeostasis, as indicated by the changes in ADP-ribosylation in numerous translational machinery; and by the emergence of proteins involved in ubiquitination (Higashi et al. 2019).

## PARPS, ADP-ribosylation, and host–pathogen interactions

ADP-ribosylation is well-known to play an important role in many host–pathogen interactions. For instance, many important bacterial toxins are ADP-ribosyltransferases (ARTs). Bacterial pathogens such as *B. pertussis*, *V. cholera*, *P. aeruginosa*, *C. botulinum*, *S. aureus*, and *E. coli* encode for ARTs that target proteins such as elongation factor 2, actin, and Rho GTPases that lead to cell death (Holbourn et al. 2006). As discussed above, mammals also encode for a diverse set of ARTs, most of which are termed PARPs, that impact infections. Here we discuss mammalian encoded PARPs that are involved in host–pathogen interactions, focusing on virus infections.

### Mammalian PARPs display several properties indicative of involvement in host–pathogen interactions

PARPs interact with pathogens in many ways, and here we describe specific cases where they either promote or restrict virus replication and the innate immune response. This discussion is summarized in Table 1 and Figure 1. However, we start by discussing characteristics that indicate an important role for PARPs in the host response to infection.

**Table 1.**
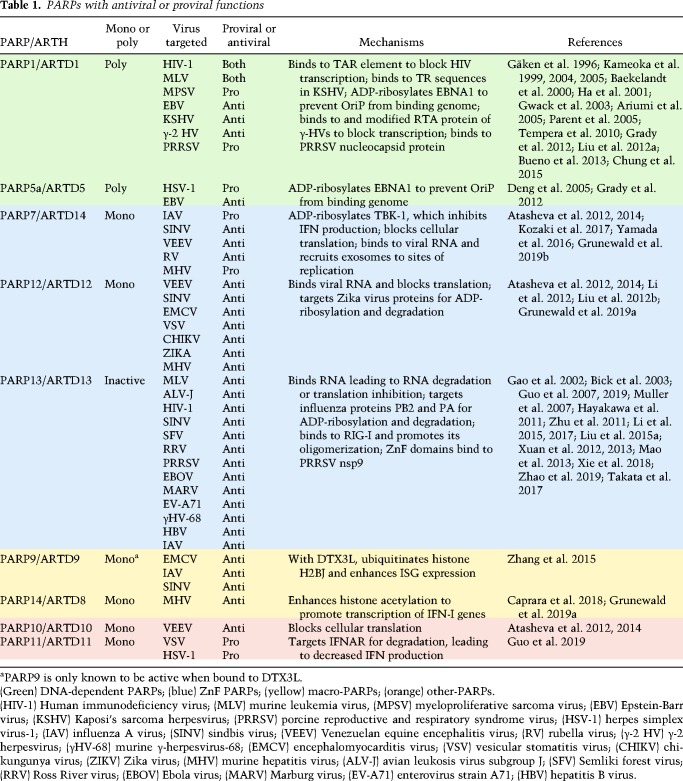
PARPs with antiviral or proviral functions

First, many mammalian PARPs are stimulated by the production of IFN (IFN-stimulated genes [ISGs]), and thus are part of the mammalian antiviral defense system (Atasheva et al. 2012; Zhang et al. 2015; Eckei et al. 2017; Li et al. 2018; Grunewald et al. 2019a). Also, several mono-ADP-ribosylating PARPs are rapidly evolving, indicating ongoing conflict with pathogens. The PARP domain of PARP13, a disordered region of PARP4, and the macrodomain(s) of the three macro-PARPs, PARP9, PARP14, and PARP15, are all under positive selection (Kerns et al. 2008; Daugherty et al. 2014). Furthermore, *Parp14* and *Parp15* have undergone multiple rounds of gene loss and duplication, which creates novel gene products needed for continual adaptation to new pathogens.

Several PARP proteins are also present in stress granules, which are important membrane-less organelles that function to restrict the translation of RNA when cells are under stress, such as during a virus infection. They are often characterized by the presence of TIA1, TIAR, and G3BP1, but are known to contain several hundred proteins (McCormick and Khaperskyy 2017). Interestingly, several proteins in stress granules are ADP-ribosylated, and PARPs, including 5a, 12, 13, 14, and 15, are known constituents of stress granules (Leung et al. 2011). It has been proposed that polyADP-ribose facilitates the concentration of RNA-binding proteins in stress granules and other nonmembranous structures and thereby promote their oligomerization (Leung 2014).

### Some virus families encode for a macrodomain protein that reverses cellular ADP-ribosylation

Several decades ago, a conserved domain was identified in all coronaviruses (CoVs), togaviruses, and hepatitis E virus that was termed the “X” domain (Gorbalenya et al. 1991; Koonin et al. 1992). These domains are structurally homologous to the nonhistone part of the macroH2A protein and are now known as macrodomains (Allen et al. 2003). Macrodomains from all three viral families bind mono- and poly-ADP-ribose, and can efficiently remove mono-ADP-ribose from proteins by hydrolysis, strongly indicating a role for ADP-ribosylation in either promoting or inhibiting the replication of these viruses (Egloff et al. 2006; Li et al. 2016). Several studies on the CoV and alphavirus macrodomains have established that this protein domain is critical for either replication or pathogenesis (Eriksson et al. 2008; Park and Griffin 2009; Fehr et al. 2015, 2016; McPherson et al. 2017). Recent studies using chikungunya virus (CHIKV) macrodomain mutants showed that macrodomain ADP-ribose binding facilitated initiation of virus replication, while hydrolase activity was essential for the amplification of replication complexes (Abraham et al. 2018). Infection with ADP-ribosylhydrolase (ARH)-deficient CoVs, including severe acute respiratory syndrome (SARS)-CoV and murine hepatitis virus (MHV), led to higher levels of IFN and other cytokines, indicating that it may block the innate immune response (Fehr et al. 2016; Grunewald et al. 2019a). The ARH-deficient MHV replicates poorly in primary macrophages, and importantly, this defect could be partially rescued by PARP inhibitors, directly indicating PARPs in the antiviral response to CoVs (Grunewald et al. 2019a). However, it remains unknown what proteins may be targeted by the viral macrodomains (for reviews, see Fehr et al. 2018; Leung et al. 2018).

### The roles of PARP1 and the Tankyrase PARPs in virus replication.

Some of the first reports of PARPs and ADP-ribosylation impacting virus infection focused on the role of PARP1 on retrovirus and HIV-1 integration and replication. Gäken et al. (1996) first demonstrated that PARP inhibitors led to reduced retroviral integration into host chromatin. They further used antisense oligonucleotides and overexpression of dominant-negative PARP1 to confirm that PARP activity is required for integration of retroviral vectors. Other groups further demonstrated the importance of PARP activity in retrovirus and HIV-1 integration into host chromosomes using siRNA transfected and PARP1-deficient cells (Ha et al. 2001; Kameoka et al. 2005). Mechanistically, it was suggested that PARP1 may help resolve a 4- to 6-bp gap in the genome produced during integration (Ha et al. 2001). PARP1 activity may also impact HIV-1 transcription and replication (Kameoka et al. 1999, 2004). However, these results have been confounded by other reports that demonstrated either no evidence that PARP1 was required for efficient HIV-1 integration or replication (Baekelandt et al. 2000; Ariumi et al. 2005), or evidence that PARP1 can repress HIV-1 or retrovirus infection (Parent et al. 2005; Bueno et al. 2013). Bueno et al. (2013) found that PARP1 inhibited retroviral infection in a chicken B lymphoblastoid cell line, while Parent et al. (2005) showed that PARP1 could bind to the transactivation response element (TAR) and inhibit HIV-1 transcription by competing with TAR for binding to p-TEF2b. The impact of PARP1 on HIV-1 infection remains controversial and is likely context-dependent.

**Figure 4.**
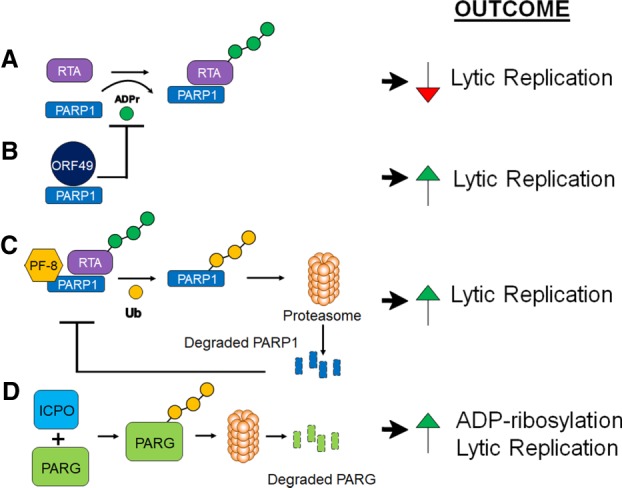
Mechanisms used by herpesviruses to affect PARylation and their impact on replication. (*A*) PARP-1 can bind to and ADP-ribosylate the γHV RTA, which inhibits its ability to initiate lytic replication. (*B*) The γHV-68 protein ORF49 binds to PARP1 and prevents it from interacting with and ADP-ribosylating RTA, which allows RTA to initiate viral gene transcription. (*C*) The KSHV and γHV-68 PF-8 proteins bind to PARP1 and target it for ubiquitination and degradation. This again prevents ADP-ribosylation of RTA, which allows it to initiate lytic replication. (*D*) The HSV-1 ICP0 protein targets PARG for ubiquitination and degradation, resulting in enhanced PARylation during infection and increased replication. (ADPr) ADP-ribose; (Ub) ubiquitin.

In addition to its role in regulating retrovirus replication, PARylation enhances and represses several other viruses. This includes herpesviruses, where PARPs have a wide range of effects. PARP1 and the tankyrase PARP5a modify the EBV protein EBNA1. PARylation of EBNA1 causes it to dissociate from the dyad symmetry elements, which restricts OriP binding and impairs the maintenance of the viral episome during latency (Deng et al. 2005; Tempera et al. 2010). PARP1 also binds to the TR sequences in KSHV, which leads to reduced viral genome levels during latency. PARP1 and the Ste-20-like kinase hKFC synergistically bind to and ADP-ribosylate/phosphorylate the γ-2 herpesvirus replication and transcription activator protein (RTA) (Fig. 4A). These interactions suppress RTA-mediated transcriptional activation and KSHV lytic reactivation (Gwack et al. 2003). Two mechanisms have been described by which γ-herpesviruses counter PARP1 activity. First, it was found that ORF49 of γHV-68 binds to PARP1, preventing it from interacting with RTA (Fig. 4B). In addition, the processivity factor of KSHV and γHV-68, PF-8, binds to and targets PARP1 for degradation, which reduces PARylated RTA and enhances virus replication (Fig. 4C; Noh et al. 2012; Chung et al. 2015). In contrast to the antiviral effects of PARPs during γ-herpesvirus infection, PARP activity seems to promote the replication of HSV-1, the prototype α-herpesvirus. PARP5a (Tankyrase-1) expression was increased and it was translocated to the nucleus during HSV-1 infection. Knockdown of both PARP5a and PARP5b resulted in a threefold to fourfold decrease in virus replication, and inhibition of their catalytic activity with XAV-939 resulted in a greater than 1-log reduction in virus replication (Li et al. 2012). To further indicate that PARP activity is important for virus replication, HSV-1 infection significantly increased PARylation. The ICP0 protein targets nuclear forms of PAR glycohydrolase (PARG), the enzyme that degrades PAR, for ubiquitination and degradation, providing a possible mechanism for the dramatic increase in PARylation during infection (Fig. 4D; Grady et al. 2012).

Finally, poly-ADP-ribosylation is implicated as having proviral activities in several viral systems. PARP inhibitors have led to greatly reduced infectivity of adenoviruses, possibly through the ADP-ribosylation of their core proteins (Déry et al. 1986). PARP inhibitors also inhibit JC virus replication (Nukuzuma et al. 2013). PARP1 binds to the porcine reproductive and respiratory syndrome virus (PRRSV) nucleocapsid protein, and again, PARP inhibitors restricted the replication of PRRSV in cell culture (Liu et al. 2015b). The nucleocapsid protein of the related coronaviruses is also ADP-ribosylated, however the impact of this modification on virus replication or pathogenesis remains unknown (Grunewald et al. 2018). Last, PARP activity is required for efficient activity of the RNA polymerases derived from multiple strains of influenza virus, indicating a potential proviral role for ADP-ribosylation during influenza infection (Bortz et al. 2011). In summary, PARylation has a variety of different functions that can both repress and enhance virus replication.

### The roles of nonenzymatic and mono-ADP-ribosylating PARPs in virus replication and the antiviral response

Nonenzymatic and mono-ADP-ribosylating PARPs have a variety of roles in promoting or inhibiting virus replication. This class of PARPs include the zinc finger (ZnF) PARPs (7, 12, and 13), the macrodomain-containing PARPs (9, 14, and 15), and several PARPs that do not fit into a specific category (4, 6, 8, 10, and 11). Here, we discuss what is known about each of these PARPs in the innate immune response to viruses.

### CCCH ZnF PARPs

ZnF PARPs contain one or more ZnF domains. These domains are small protein motifs that enable these PARPs to bind RNA. All three ZnF PARPs use this domain to interact with viral RNA and inhibit either translation or degrade viral RNA, though the specific RNA sequence that each PARP binds to is likely unique. In addition, all three ZnF PARPs use either the enzymatic or nonenzymatic functions in their PARP domain to impact the innate immune response or virus replication.

#### PARP13 (ZnF antiviral protein)

PARP13, or ZnF antiviral protein (ZAP), was one of the first PARPs identified to have antiviral activities when, in a screen for antiviral ISGs, it was found to potently inhibit murine leukemia virus (MLV) replication (Gao et al. 2002). Somewhat surprisingly, ZAP lacks the triad motif (H-Y-E) needed for catalytic activity and has no auto-ADP-ribosylating activity (Kleine et al. 2008). As such, most of its antiviral activity is independent of ADP-ribosylation. Since its discovery, ZAP has been shown to inhibit the replication of several viral families, including retroviruses, alphaviruses, filoviruses, picornaviruses, herpesviruses, arteriviruses, orthomyxoviruses, flaviviruses, and hepatitis B virus (Bick et al. 2003; Muller et al. 2007; Zhu et al. 2011; Wang et al. 2012; Xuan et al. 2012; Mao et al. 2013; Xuan et al. 2013; Li et al. 2015, 2015a; Chiu et al. 2018; Xie et al. 2018; Zhao et al. 2019). ZAP is transcribed into four different isoforms, with ZAPL and ZAPS being the most studied (Li et al. 2019). ZAPL contains the inactive PARP or catalytic domain, while ZAPS does not. ZAPL tends to have greater antiviral activity, and this may be due, at least in part, to prenylation of the PARP domain (Charron et al. 2013; Schwerk et al. 2019). In addition to the PARP domain, ZAP contains four ZnF-binding domains and a single WWE domain. ZAP uses its ZnF-binding domains to bind to viral RNA and recruits both the poly(A)-specific ribonuclease (PARN) and the RNA exosome to degrade the viral RNA (Guo et al. 2007). It also inhibits translation by blocking the interaction between eIF4G and eIF4A, and its ability to block translation is required for it to degrade RNA. In addition, ZAP antiviral activity is enhanced by the ubiquitin ligase activity of TRIM25. TRIM25 binds to ZAP and ubiquitinates unknown proteins to enhance the antiviral activity of ZAP (Li et al. 2017; Zheng et al. 2017).

ZAP targets HIV RNAs for degradation, and prefers to target CG dinucleotides (Takata et al. 2017). Consistent with this, an HIV-1 mutant with an increased CG content replicated very poorly in MT4 cells, but that replication was restored in ZAP-deficient cells. In addition, ZAP targets the 3′ UTR of Japanese encephalitis virus (JEV), which contains a high CG content (Chiu et al. 2018). Interestingly, it appears many viruses maintain a low CG dinucleotide level, and the level of ZAP sensitivity of several viruses mildly correlates with their CG dinucleotide content (Takata et al. 2017). However, ZAP sensitivity of a panel of alphaviruses does not correlate with the CG dinucleotide content found in their genome or individual viral genes, suggesting that the CG dinucleotide motif is not the only determinant for ZAP recognition (Li et al. 2019). The localization of ZAP to stress granules also appears to be functionally important for its antiviral activity against alphaviruses, as ZAP mutants that do not localize to SGs are unable to block SINV replication (Law et al. 2019). In vivo, *Zap* knockout (*Zc3hav1^−/−^*) mice showed enhanced replication of SINV in 10-d-old mice as expected (Kozaki et al. 2015). Though surprisingly, in 23-d-old weanling pups it was shown that a neurovirulent strain of SINV can use ZAP to decrease its replication in initially infected cells in vivo such that it prevents immune recognition, allowing the virus to spread to the central nervous system (CNS) and promote disease (Wang et al. 2016).

In addition, different studies have found conflicting results regarding the ability of ZAP to impact the innate immune response. It was originally found that ZAPS, but not ZAPL, potentiates RIG-I-dependent type I interferon (IFN-I) production in human cells by binding to RIG-I via its ZnF domains and promoting its oligomerization (Fig. 1A; Hayakawa et al. 2011; Chiu et al. 2018). However, ZAP does not appear to enhance RIG-I-dependent IFN-I production in mouse cells (Lee et al. 2013). More recent data indicate that ZAPS may also reduce IFN mRNA by binding to the 3′ UTR of IFN mRNA and targeting it for degradation (Fig. 1A; Schwerk et al. 2019). It is conceivable that ZAP-S uses both functions but in a context-dependent manner.

**Figure 5.**
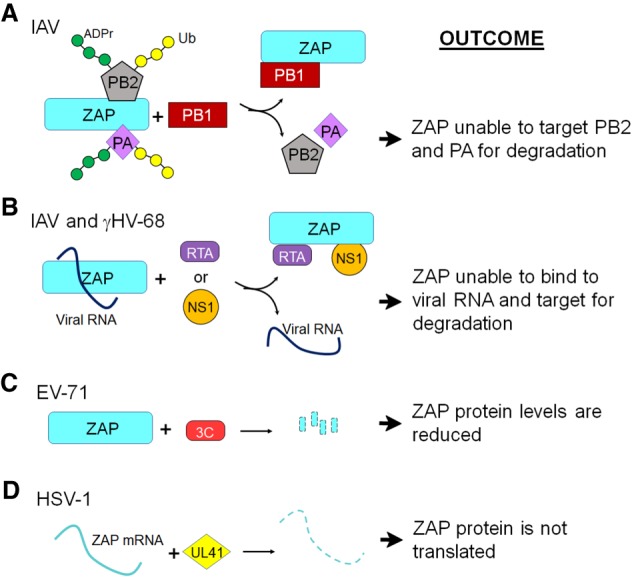
Viral mechanisms of ZAP antagonism. (*A*) IAV protein PB1 binds to ZAP, which prevents its interaction with the PA and PB2 proteins that otherwise would lead to PARylation, ubiquitination, and degradation of these proteins. (*B*) IAV NS1 and γHV-68 RTA proteins interact with ZAP, preventing its association with viral RNA. (*C*) The EV-71 3C protease cleaves ZAP to prevent it from accumulating. (*D*) HSV-1 UL41 protein cleaves ZAP mRNA to prevent its translation. (ADPr) ADP-ribose; (Ub) ubiquitin.

While ZAPL does not contain an active PARP domain, in some cases it is required for the ADP-ribosylation of proteins. For instance, the C terminus of ZAPL binds to the influenza virus polymerase proteins PB2 and PA, which causes their subsequent poly-ADP-ribosylation, ubiquitination, and degradation (Liu et al. 2015a). It is unknown which PARP and E3 ubiquitin ligase mediates these effects. Knockdown of ZAPL modestly increased influenza virus replication in cell culture, though it is not clear whether this is due to its ability to bind and target PB2 and PA for degradation. Interestingly, this ZAPL activity was countered by the PB1 protein, which bound to ZAPL preventing the ubiquitination of PB2 and PA, demonstrating that the virus has evolved ways to neutralize the antiviral activity of ZAP (Fig. 5A). In addition to PB1, several other viral proteins have been found to counter ZAP using multiple mechanisms (Fig. 5B–D). Influenza A NS1 prevents ZAP-S from binding to its target RNA (Tang et al. 2017), γHV-68 RTA disrupts the intermolecular interaction of ZAP (Xuan et al. 2013), HSV-1 UL41 degrades ZAP mRNA (Su et al. 2015), and, finally, the enterovirus (EV)-71 3C protease cleaves ZAP protein (Xie et al. 2018). Due to its broad-spectrum antiviral activity, it is likely there are even more viral proteins that function to counter ZAP.

#### PARP12

PARP12 is a mono-ADP-ribosyltransferase and has four or five N-terminal CCCH-type zinc finger (ZnF) domains that are important for RNA binding, one or two WWE domains in the middle of the protein that are important for ADP-ribose binding, and a PARP domain at the C terminus, which provides the protein with mono-ADP-ribosylating activity (Welsby et al. 2014). There are two splice forms of *PARP12* mRNA, PARP12L, and PARP12S (Atasheva et al. 2012). PARP12L contains both the ZnF domains and the PARP catalytic domain, while PARP12S has the ZnF domains but lacks the PARP catalytic domain.

As described above, PARP12 translocates to cytoplasmic stress granules upon cell stress (Leung et al. 2011; Welsby et al. 2014). PARP12 initially localizes to the *trans*-Golgi network (TGN) and translocates to stress granules during stress stimuli in several different cell types. This activity has been linked to the ZnF domains, as mutations in the ZnF domains abrogated the ability of PARP12 to move to stress granules (Welsby et al. 2014). The translocation of PARP12 may also depend on PARP1 acting as a stress sensor in the nucleus, as an increase in unconjugated PAR is a key factor that promotes the recruitment of PARP12 to stress granules (Catara et al. 2017). The translocation of PARP12 to stress granules is reversible, as it relocates back to the Golgi once the stress is relieved.

The antiviral role of PARP12 was first described in an overexpression screen, where it mildly inhibited the replication of both VSV and MHV-68 (Liu et al. 2012b). Shortly after this, another study found that PARP12 was differentially expressed in cells that cleared VEEV replication compared with those that were persistently infected (Atasheva et al. 2012). Further analysis showed that PARP12L, but not PARP12S, expression from a VEEV replicon or virus restricts VEEV replication, as well as several other viruses including Sindbis virus (SINV), encephalomyocarditis virus (EMCV), vesicular stomatitis virus (VSV), Rift Valley fever virus (RVFV), and chikungunya virus (CHIKV). Subsequently, this group showed that PARP12 strongly inhibited both cellular and viral translation (Atasheva et al. 2014). Immunoprecipitation with PARP12 identified several ribosomal proteins and translation and elongation factors, indicating that PARP12 interacts with ribosomes. Interestingly, PARP12 required its enzymatic activity to block translation, but not to inhibit virus replication, indicating that PARP12 may use distinct mechanisms to block virus replication and cellular translation.

Additionally, PARP12 was identified in a screen for ISGs that inhibit Zika virus (ZIKV) (Li et al. 2018). Using both knockout cells and overexpression, the authors showed that PARP12 was both necessary and sufficient for the inhibition of ZIKV replication. PARP12 was required for the ADP-ribosylation, ubiquitination, and subsequent degradation of ZIKV NS1 and NS3 proteins. This activity did not require either the ZnF or WWE domains of PARP, but did require its catalytic domain, as an inactive catalytic domain reversed the regulatory effects of PARP12 on viral protein degradation. Interestingly, NS1 and NS3 appeared to be poly-ADP-ribosylated, indicating that PARP12 may work with another PARP to mediate the poly-ADP-ribosylation of these proteins. PARP12 also has a role in the restriction of coronavirus (CoV) replication, as siRNA knockdown of PARP12 partially restored replication of MHV lacking the ADP-ribosylhydrolase (ARH) activity of the CoV macrodomain (Grunewald et al. 2019a). The mechanism used by PARP12 to restrict MHV replication remains unknown. Finally, PARP12 was also shown to enhance NF-κB signaling, possibly by interacting with TRIF (Fig. 1A; Welsby et al. 2014).

#### PARP7 (TiPARP)

PARP7, also known as tetrachlorodibenzo-p-dioxin (TCDD)-inducible poly-ADP-ribose polymerase (TiPARP), has a single ZnF domain that mediates RNA binding. It also has a WWE domain and a PARP catalytic domain capable of mono-ADP-ribosylation (Kozaki et al. 2017).

Like PARP12, TiPARP was also shown to block VEEV replication and inhibit cellular translation when transduced into cells by a VEEV replicon (Atasheva et al. 2014). In a separate study, siRNA knockdown of TiPARP in U373 human astrocyte cells led to increased replication of SINV and rubella virus replication (Kozaki et al. 2017). The increase in SINV replication was also demonstrated in *TiParp*^−/−^ mice. TiPARP-mediated inhibition of SINV was dependent on its ZnF domain, which binds to SINV RNA and recruits RNA degradation factors to sites of viral replication. This suggests that TiPARP recognizes specific virus RNAs for degradation, however the target specificity of TiPARP is still unknown.

TiPARP also has proviral effects in addition to its antiviral functions. TiPARP negatively regulates the type I IFN response by ADP-ribosylating TBK1 (Fig. 1A; Yamada et al. 2016). The ADP-ribosylation of the kinase domain of TBK1 suppresses IFN production. It was suggested that negative regulation of IFN production by TiPARP may help protect the cell from the harmful effects of type I IFN. The same study found that the loss of TiPARP led to decreased IAV replication, which strongly correlated with increased IFN-I production. Our group has also found that siRNA knockdown of TiPARP led to decreased replication of MHV and increased IFN-I production, further indicating TiPARP as a proviral factor for some viruses (Grunewald et al. 2019a,b). However, it remains unclear whether the ability of TiPARP to enhance MHV or IAV replication is tied to its ability to block the IFN-I response.

### Macrodomain-containing PARPs

The macro PARPs contain two (PARP9/PARP15) or three (PARP14) macrodomains that mediate binding to ADP-ribose, as described above. While some macrodomains can cleave ADP-ribose from a substrate, it is likely that macrodomains within these PARP proteins only bind ADP-ribose. All three macro PARPs are rapidly evolving (Daugherty et al. 2014), and PARP15 has been identified in stress granules (Leung et al. 2011); however, direct evidence of their involvement in virus infections is limited. No study has identified a role for PARP15 in modulating virus infection or the innate immune response, and thus it will not be discussed further.

#### PARP9 (BAL1)

PARP9 was originally termed B-aggressive lymphoma 1 gene (BAL1) as it was identified as a risk factor for large diffuse B-cell lymphomas (Aguiar et al. 2005). It is catalytically inactive, at least when expressed by itself, but can ADP-ribosylate ubiquitin in the presence of an E3 ubiquitin ligase, DTX3L (Yang et al. 2017). As described above, PARP9 promotes STAT1 phosphorylation, proinflammatory gene expression, and differentiation into M1-like macrophages (Iwata et al. 2016). In the antiviral response, PARP9 expression in malignant B-cell lymphoma lines can lead to widespread induction of ISG expression (Juszczynski et al. 2006). Zhang et al. (2015) found that the complex of PARP9 and DTX3L attached to STAT1 to mediate the hyper-responsiveness of a mutant STAT1 protein (STAT1-CC). The PARP9–DTX3L complex ubiquitinates histone proteins, most notably H2BJ, which led to chromatin remodeling and enhanced expression of at least a subset of ISGs (Fig. 1B). This interaction was necessary and sufficient to inhibit the replication of multiple viruses, including EMCV, IAV, and SINV. The PARP9–DTX3L complex also ubiquitinates the EMCV 3C protease, which led to its degradation, but this effect was mostly, if not completely, due to DTX3L activity.

#### PARP14 (CoaST-6)

PARP14 was originally identified as Collaborator of STAT6 (CoaST6) (Goenka and Boothby 2006). It has a range of effects on cell physiology and immunity that were largely anti-inflammatory (Cho et al. 2011, 2013; Barbarulo et al. 2013; Vyas et al. 2013; Iansante et al. 2015; Iwata et al. 2016; Krishnamurthy and Kaplan 2016). In the antiviral response, PARP14 instead is required to enhance IFN-I production in RAW cells (transformed peritoneal macrophages) following LPS treatment (Caprara et al. 2018), in primary macrophage cells during CoV infection, and following treatment of human A549 cells with poly(I:C) (Grunewald et al. 2019a). Following LPS treatment, PARP14-deficient cells showed similar levels of IRF-3 phosphorylation and nuclear translocation, but had reduced levels of Pol II recruited to the promoters of IRF-3-dependent genes (Caprara et al. 2018). There was also a dramatic reduction in H3K27 acetylation, a known marker of active promoters and enhancers (Fig. 1A). It is unclear whether this function of PARP14 is dependent on its catalytic activity. It is also not yet known whether PARP14 has the same function in the cellular response to virus infection or poly(I:C). These two studies also found that PARP14 was required to restrict the replication of *S. typhimurium* and an ARH-deficient MHV, though it is not known whether the ability of PARP14 to inhibit these pathogens is tied to its role in up-regulating IFN production or whether these are distinct functions of PARP14. It is important to note that these experiments were done with undifferentiated or M0 macrophages, while other studies where PARP-14 was found to have anti-inflammatory functions used M1 or M2 macrophages, differentiated by further IFN-γ or IL-4 treatment. This suggests that PARP14 function is likely context-dependent.

### Other PARPs

The final class of PARPs do not fit into any of the other defined classes of PARPs, have no similar domains other than the PARP domain, and are thus simply termed “other PARPs.” These PARPs include PARPs 4, 6, 8, 10, 11, and 16. PARP6 and PARP8 have no defined domains besides their PARP domain, and neither have a known role in the immune response. PARP4, while rapidly evolving as described earlier, has not been reported to have any direct antiviral or proviral activity. PARP16 promotes ER stress responses by ADP-ribosylating IRE1α and PERK (Jwa and Chang 2012), and also ADP-ribosylates Karyopherin β1, indicating a potential role in nuclear transport (Di Paola et al. 2012). However, it also has no known antiviral activities or impact on the innate immune response. Here we focus on PARP10 and PARP11.

#### PARP10

PARP10 contains both an RNA recognition motif (RRM), nuclear import and export signals, and two ubiquitin-interacting motifs (UIM) in addition to its catalytic domain (Verheugd et al. 2013) and is highly up-regulated by IFN (Eckei et al. 2017; Grunewald et al. 2019a). Along with PARP12 and PARP7, it inhibits VEEV replication and blocks protein translation when expressed from a VEEV replicon (Atasheva et al. 2014). PARP10 also blocks NF-κB signaling and the production of proinflammatory cytokines (Verheugd et al. 2013). Mechanistically, the UIM of PARP10 interacts with K63-linked ubiquitin chains and NEMO. PARP10 ADP-ribosylates NEMO and prevents its polyubiquitination, which ultimately blocks NF-κB from translocating to the nucleus to activate gene expression (Fig. 1A). It remains unknown whether this function of PARP10 impacts host–pathogen interactions or whether it functions in other contexts.

#### PARP11

PARP11 is the second smallest PARP protein (PARP16 is the smallest), with only a single WWE domain in addition to its ART domain, and is also highly up-regulated by IFN (Grunewald et al. 2019a). Recently, PARP11 was shown to block IFN signaling by ADP-ribosylating the E3 ubiquitin ligase β-transducin repeat-containing protein (β-TrCP), which lead to the ubiquitination and degradation of the interferon α/β receptor (IFNAR) (Fig. 1B; Guo et al. 2019). siRNA silencing of PARP11 or treatment with rucaparib, a pan-PARP inhibitor used in advanced ovarian cancer, inhibited the replication of VSV and HSV-1. While normally known to inhibit PARP1/2, at the concentrations of drug used in this study rucaparib appeared to preferentially target PARP11. Interestingly, following infection in vivo, rucaparib enhanced IFN-I signaling, reduced VSV replication in multiple organs, and led to better outcomes for the mice infected with either VSV or HSV-1. These data indicate that PARP11-specific inhibitors could be a useful means of treating specific viral infections.

In summary, several of the nonenzymatic or mono-ADP-ribosylating PARPs are potent antiviral proteins that are able to inhibit viruses from several different viral families. However, some do contain activities that promote virus replication. While some mechanisms are known, including blocking translation, degrading RNA, and targeting viral or host proteins for ubiquitination and degradation, many mechanisms are still unknown. However, recent reports are making it clear that in many, but not all cases, ADP-ribosylation is tied to protein homeostasis, either through mediating translation or ubiquitination-dependent protein degradation. In addition, several studies have identified multiple points where the innate immune response is modulated by PARPs and ADP-ribosylation (Fig. 1). The identification of similar and potentially novel processes mediated by PARPs in virus infections will likely be uncovered in the near future.

## Final remarks

With the advent of new mass spectrometry techniques and improved tools for detecting ADP-ribose, the last decade has seen an explosion in our understanding of how PARPs and ADP-ribose impact not just immunity, but biology in general. This technological development is exponential, which will further facilitate PARP research in the future. PARP inhibitors are being tested in the clinic for chemotherapy, so it is likely that PARP inhibitors or agonists could be useful for treating immune disorders as well. However, developing PARP-specific inhibitors or agonists will be challenging. There is still a long way to go before we fully understand how PARPs function both in cell culture and in vivo to target them for the treatment of infections or immune diseases. Additional PARP-deficient animals and specific inhibitors are needed to gain a better knowledge of how PARPs impact pathogenesis from infection or immune-mediated diseases. While PARPs are structurally and functionally distinct, specificity and off-target effects of PARP inhibitors remain incompletely understood; thus, further characterization of each compound is necessary.

Accumulating clinical and scientific evidence supports a theory that inflammation promotes various global health threats such as myocardial infarction. However, mechanisms of macrophage activation, for instance, remain obscure. Recent understanding that macrophages are highly heterogeneous has added to the complexity of inflammation. What is the role of each PARP in inflammation via an enzymatic activity-dependent or independent mechanism? What is a specific function of each domain in macrophage activation? Furthermore, additional roles for PARPs in the antiviral response are likely to exist, especially for PARPs that are under strong positive selection pressure, but have yet to be associated with a specific biological activity. In several cases the mechanism used by PARPs to inhibit specific viruses remain unknown. It is also intriguing that several IFN-induced PARPs have been shown to enhance virus replication. Could this be a mechanism by the host to maintain a minimal amount of virus replication in vivo so that the immune system can be appropriately activated? The answers to these and many other questions will be of great interest to PARP researchers, immunologists, and microbiologists as they are likely to uncover unique cellular processes regulated by ADP-ribosylation that could lead to the identification of novel therapeutic targets for infections or immune-mediated diseases.
